# Cardiac Proteome Profiling in Ischemic and Dilated Cardiomyopathy Mouse Models

**DOI:** 10.3389/fphys.2019.00750

**Published:** 2019-06-18

**Authors:** Danbo Lu, Yan Xia, Zhangwei Chen, Ao Chen, Yuan Wu, Jianguo Jia, Aijun Sun, Yunzeng Zou, Juying Qian, Junbo Ge

**Affiliations:** Department of Cardiology, Shanghai Institute of Cardiovascular Diseases, Zhongshan Hospital, Fudan University, Shanghai, China

**Keywords:** heart failure, ischemic cardiomyopathy, dilated cardiomyopathy, proteomics, metabolism

## Abstract

Heart failure (HF) is a worldwide pandemic with an unacceptable high level of morbidity and mortality. Understanding the different pathophysiological mechanisms will contribute to prevention and individualized therapy of HF. We established mouse models for ischemic cardiomyopathy (ICM) and dilated cardiomyopathy (DCM) by inducing myocardial infarction (MI) and Coxsackievirus B3 infection, respectively. Isobaric tags for relative and absolute quantitation and liquid chromatography coupled with tandem mass spectrometry technology was used to identify the protein expression profiles in control and failing hearts. A total of 1,638 proteins were identified and compared in this proteomics analysis. Among them, 286 proteins were differently expressed. Gene ontology, KEGG pathway and ingenuity pathway analysis was performed to systematically assess the potential connections of the differentially expressed proteins to biological functions. Compared with control group, the differentially expressed proteins derived from the hearts of ICM and DCM mice were partially similar and mainly modulated in oxidative phosphorylation, metabolism and protein folding pathways. Moreover, difference still existed, the differentially expressed proteins between DCM and ICM hearts were significantly modulated in oxidative phosphorylation, metabolic and AMPK signaling pathways. Confirmatory western bolt analysis demonstrated that SDHB was down-regulated in both ICM and DCM hearts, while UQCRQ, GLUT4 and adiponectin were up-regulated in ICM hearts. Adenosine triphosphate (ATP) concentration significantly decreased in both DCM and ICM hearts. The protein expression of phospho-AMPKα decreased significantly in DCM hearts, but increased in ICM. In summary, oxidative phosphorylation, cardiac metabolism, and protein folding play critical roles in the pathogenesis of HF. The diverse changes in protein expression profiles between failing hearts induced by either MI or CVB3 infection demonstrated the heterogeneity of HF. Understanding the differences in proteome profiles could offer more precise therapeutic options for HF.

## Introduction

Heart failure (HF) is a worldwide pandemic with an unacceptable high level of morbidity and mortality and results in an enormous medical and socio-economic burden (Benjamin et al., 2018). Chronic HF is characterized by progressive alterations in cardiac structure and function. However, the molecular mechanism of these alterations is yet to be fully deciphered. HF is a highly heterogeneous disease that can result from a multitude of factors (Neubauer, 2007). Dilated cardiomyopathy (DCM) and ischemic cardiomyopathy (ICM) accounts for the majority of HF (Benjamin et al., 2018).

Myocarditis is an important reason that results in DCM. Virus infection is the most common pathogen that induces myocarditis (Corsten et al., 2012; Sagar et al., 2012; Weintraub et al., 2017). Viral genomes can be detected in cardiac tissue in 20–67.4% of DCM patients (Bowles et al., 2003; Kühl et al., 2005). Coxsackievirus B3 (CVB3) is the dominant etiological agent that induces acute and chronic viral myocarditis (Bowles et al., 2005; Esfandiarei and McManus, 2008; Weintraub et al., 2017). Both direct virus- and immune-mediated injuries contribute to the development of myocarditis and ultimately to DCM. CVB3 induced experimental murine models for HF have been widely used to investigate the molecular pathology of DCM (Esfandiarei and McManus, 2008). In addition, ICM results in more than 60% of all patients developing systolic congestive heart failure. Myocardial infarction (MI) and subsequent post-infarct remodeling are major etiologies of ICM (Sutton and Sharpe, 2000).

While HF is the common manifestation of DCM and ICM, they differ in their etiology and pathogenesis. Understanding the different pathophysiological mechanisms will help contribute to prevention and individualized patient therapy of heart failure. The changes in protein levels in myocardial tissue can reflect these diverse changes. Isobaric tags for relative and absolute quantitation (iTRAQ), a liquid chromatography coupled with tandem mass spectrometry technology (LC-MS) based proteomics approach, has been demonstrated to provide accurate mass measurements to determine changes in protein levels. In addition, systemic bioinformatic analysis has become increasingly important to monitor and assess changes in protein levels. Previous studies using proteomic approaches have demonstrated several novel pathophysiological mechanisms for cardiovascular diseases, including ICM (Xiang et al., 2011; Liu et al., 2014), DCM (Nishtala et al., 2011), atherosclerosis (Herrington et al., 2018), diabetic cardiomyopathy (Edhager et al., 2018), and coronary microembolization (Chen et al., 2018). To date, no studies have been performed to compare the proteomic profiles of ICM and DCM in murine models for HF.

The present study used a proteomic approach to investigate changes in tissue protein expression in the left ventricle from murine models with ICM and DCM.

## Materials and Methods

### Animals

Mice were purchased from LingChang BioTech Co. Ltd. (Shanghai, China). Eight-week-old C57BL/6 male mice were randomly divided into three groups (n = 10 per group): the NC group (sham-surgery and saline injection), DCM group (sham-surgery and CVB3 injection) and ICM group (MI-surgery and saline injection). 1 × 10^5^ TCID_50_/ml × 0.3 ml CVB3 was intraperitoneally injected into each mouse in the DCM group (Li et al., 2016). MI and sham-surgery was performed in 16-week-old mice by permanent ligation of the left anterior descending coronary artery, as previously described (Gao et al., 2010). All mice were housed in SPF conditions for 12 weeks (4 weeks after MI and 12 weeks after CVB3 injection) before being euthanized. This study was carried out in accordance with the Guide for the Care and Use of Laboratory Animals published by the US National Institutes of Health (NIH Publication No. 85-23, revised 1996). The protocol was approved by the Animal Care and Use Committee of Fudan University Zhongshan Hospital.

### Echocardiography

Four weeks after MI and 12 weeks after CVB3 administration, transthoracic echocardiography was performed using the Vevo770 ultrasound system (VisualSonics, Canada). The mice were anesthetized with 2% isoflurane (Baxter, Denmark) and placed in a supine position on a heated platform. Left ventricular ejection fraction (LVEF), left ventricular end-diastolic diameter (LVEDD), left ventricular end-systolic diameter (LVESD), and fractional shortening (FS) were measured and calculated from the M-mode images. All measurements were averaged using five consecutive cardiac cycles.

### Sample Collection and Histological Analysis

After euthanasia, mice hearts were carefully harvested. Tissues were washed with phosphate buffer saline to remove blood and then frozen in liquid nitrogen and stored at -80°C for future analysis. Additional tissues were fixed in 4% paraformaldehyde, embedded in paraffin, cut in 5 mm sections and stained with hematoxylin and eosin (HE) to evaluate heart morphology. Histological images were obtained using an Olympus BX-51 light microscope (Olympus America Inc., United States).

### Protein Preparation and iTRAQ Labeling

Three heart samples from each group with typical echocardiographic changes (NC, normal manifestations; DCM, dilated ventricle and reduced LVEF; ICM, impaired anterior wall motion, dilated left ventricle and reduced LVEF) were randomly selected for proteomic analysis. Fifty milligrams from each sample were homogenized in liquid nitrogen and then lysis buffer [30% sucrose, 0.5M Tris-HCl, 50 mM EDTA, 20 mM DTT, 0.1M KCl, 2% SDS, 1 × Protease Inhibitor Cocktail (Thermo Fisher Scientific, United States)] was added to the samples. Cell lysis was performed by sonication on ice for 1 h followed by centrifugation at 4,000 g for 30 min at 4°C. Tris-saturated phenol (pH 7.5) was then added to the tube and vortexed for 30 min before being centrifuged at 4,000 g for 15 min at 4°C. The organic phase was precipitated using five volumes of prechilled acetone at −20°C overnight. Protein concentration was determined using the Bradford assay method. The proteins were then digested using the protocol previously described (Wiśniewski et al., 2009). After digestion and cleanup, the peptide mixture was labeled using the iTRAQ 8 Plex labeling kit (AB Sciex, United States) following the manufacturer’s instructions. The labeled peptide samples were then pooled and lyophilized using a vacuum concentrator.

### LC-MS/MS

Peptides were re-dissolved in solvent A (A:2%ACN + 0.5% acetic acid) and separated through the nanoflow CHIP-LC system C. The peptides were auto-loaded into the Dionex Acclaim Pepmap100 C18 trap column (2 cm × 100 μm, 5 μm; Thermo Fisher Scientific, United States), and subsequently eluted into a C18 analytical column (75 μm × 25 cm, 2 μm; Thermo Fisher Scientific, United States) for gradient elution from 4% solvent B (B: 98%ACN + 0.5% acetic acid) to 100% solvent B in 150 min. LC-MS/MS was performed using the TripleTOF mass spectrometer (AB Sciex, United States).

### Data Processing

Tandem mass spectra was processed using the PEAKS Studio version 8.5 (Bioinformatics Solutions Inc., Canada). PEAKS DB was formatted to search the UniProt-Mus musculus database (52,194 entries, ver 201711) with trypsin as the digestion enzyme. PEAKS DB was searched with a fragment ion mass tolerance of 0.05 Da and a parent ion tolerance of 20 ppm. Carbamidomethylation (C) and iTRAQ 8plex (K, N-term) were specified as the fixed modification. Oxidation (M), Deamidation (NQ), Acetylation (Protein N-term), were specified as the variable modifications. Peptides were filtered using 1% false discovery rate (FDR) and 1 unique. ANOVA was used for peptide and protein abundance calculation. Normalization was performed by averaging the abundance of all peptides. Medians were used for averaging. Fold change was calculated as a ratio of relative protein abundance. Differentially expressed proteins were filtered if their fold change was greater than 1.5 or smaller than 0.67 and had at least two unique peptides.

### Bioinformatics Analysis

Hierarchical cluster analysis was performed using the pheatmap package.^1^ Blast2GO version 4 was used for functional annotation and GO analysis. The whole protein sequence database was analyzed by BlastP using the entire database and mapped, and then annotated using the gene ontology database. Statistically altered functions of the differently expressed proteins was calculated using Fisher’s exact test in BLAST2GO. Pathway analysis was performed using the KEGG database,^2^ *E*-value was set as 1 × 10^–5^ when conducting BLAST. The significantly altered GO functions and KEGG pathways with a FDR <0.05 were identified. Protein-protein interaction networks were constructed using STRING v10,^3^ network edges was set as “Experiments, Databases, Co-expression, Neighborhood, Gene Fusion, Co-occurrence,” interaction score was set as “default.” To further appreciate the biological significance of the differentially expressed proteins, ingenuity pathway analysis (IPA) was used to analyze canonical pathways and their relationships.

### Western Blot Analysis

Validation of LC-MS/MS results of the differentially expressed proteins was performed by western blot analysis. Total protein (40 μg) was run on a 6–12% sodium dodecyl sulfate–polyacrylamide gel and then transferred to polyvinylidene fluoride (PVDF) membranes (Millipore, United States). Membranes were blocked for 1 h in 5% skim milk in TBS-T buffer and incubated overnight at 4°C with the following antibodies: GLUT4, adiponectin, UQCRQ, SDHB, GAPDH, phospho-AMPKα (T172 and T183), and total-AMPKα. After overnight incubation, secondary antibody was incubated for 1.5 h. Antigen–antibody complexes were detected using an enhanced chemiluminescence kit (Thermo Fisher Scientific, United States). The density of the detected protein bands were quantified using Image Lab 6.0 software (Bio-Rad, United States).

### Measurement of Adenosine Triphosphate (ATP) Concentration in the Heart

Heart mitochondria were isolated using a Mitochondria Isolation Kit (Sigma-Aldrich). Briefly, heart tissues were isolated and pretreated with extraction buffer containing 0.25 mg/ml trypsin. Albumin with a final concentration of 10 mg/ml was used to quench the proteolytic reaction. After homogenization, supernatant liquid was selected after a centrifugation at 600 × *g* for 5 min. Finally, supernatant was centrifuged at 11,000 × *g* for 10 min and the mitochondria pellet was suspended in storage buffer. ATP concentration was measured using a ATP Assay Kit (Beyotime Biotechnology). Briefly, mitochondria pellet was treated using the lysis buffer. Then samples and ATP standard were added into the detector tube containing ATP Assay working solution. Relative light unit was measured with a luminometer. ATP concentration was calculated according to the standard curve.

### Statistical Analysis

Data with normal distribution was presented as mean ± SEM. Differences between two groups were determined using Student’s *t*-test. One-way ANOVA was used to compare differences among the groups. *P*-values < 0.05 were considered statistically significant. All data analyses were performed using GraphPad Prism version 5.0 software and SPSS 20.0 statistical software.

## Results

### Echocardiography Changes Induced by CVB3 and MI

**FIGURE 1:**
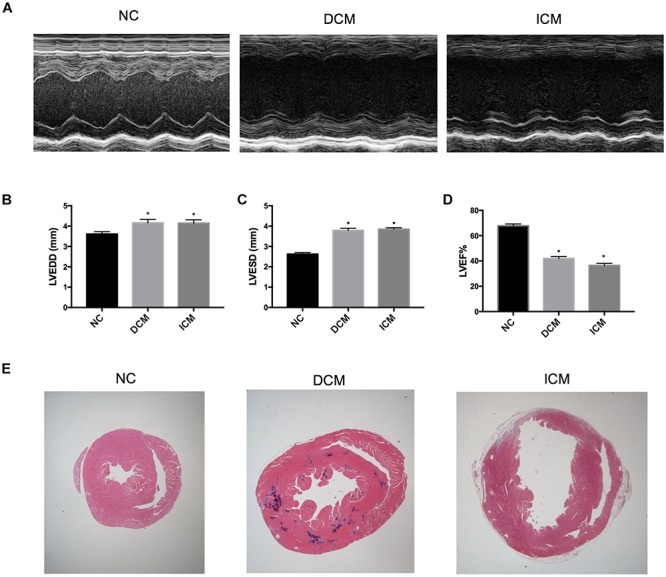
Echocardiography and histological changes in failing hearts induced by CVB3 and myocardial infarction. **(A)** The representative M-mode echocardiogram for normal, DCM and ICM hearts. The changes of **(B)** LVEDD, **(C)** LVESD, and **(D)** LVEF in failing hearts. **(E)** The representative hematoxylin and eosin stain of heart sections from NC, DCM and ICM. *n* = 10 per group. ^*^P < 0.05 vs. NC group. NC, control group; DCM, dilated cardiomyopathy; ICM, ischemic cardiomyopathy; LVEDD, left ventricular end-diastolic diameter; LVESD, left ventricular end-systolic diameter; LVEF, left ventricular ejection fraction.

Echocardiography demonstrated that 12 weeks after CVB3 injection, mice in the DCM group had dilation of the left ventricle and reduced left ventricular systolic function (Figure 1A). This was similar to symptoms observed in human patients with DCM. Compared to mice in the control group, mice injected with CVB3 had significantly elevated LVEDD and LVESD while LVEF was significantly decreased (*P* < 0.05) (Figures 1B–D). Four weeks after MI, mice in the ICM group had severely impaired anterior wall motion (Figure 1A), significantly increased LVEDD and LVESD, and decreased LVEF (*P* < 0.05) (Figures 1B–D). The detailed echocardiography data were presented in Table 1.

**TABLE 1:** Echocardiography data of DCM, ICM and NC groups.

**Echocardiography parameters**	**NC *n* = 10**	**DCM *n* = 10**	**ICM *n* = 10**
LVEDD (mm)	3.61 ± 0.12	4.16 ± 0.17	4.15 ± 0.17
LVESD (mm)	2.62 ± 0.08	3.79 ± 0.10	3.84 ± 0.09
LVEF (%)	67.75 ± 1.49	41.99 ± 1.49	36.44 ± 1.57

*LVEDD, left ventricular end-diastolic diameter; LVESD, left ventricular end-systolic diameter; LVEF, left ventricular ejection fraction; DCM, dilated cardiomyopathy; ICM, ischemic cardiomyopathy; NC, control group.*

### Cardiac Morphological Changes Induced by CVB3 and MI

HE-stained heart tissues samples from DCM mice 12 weeks post-CVB3 injection had a dilated phenotype with a significant thinning of the ventricular walls compared to non-infected controls compared with non-infected controls (Figure 1E). In addition, serious myocardium inflammation infiltration was observed in the abundant inflammatory lesions distributed within the heart tissues. Four weeks post-MI, the infarcted myocardial regions became fibrotic and were accompanied by cardiomyocyte enlargement and inflammation infiltration along the peripheries of the infarct area (Figure 1E).

### Proteomic Analysis

**FIGURE 2:**
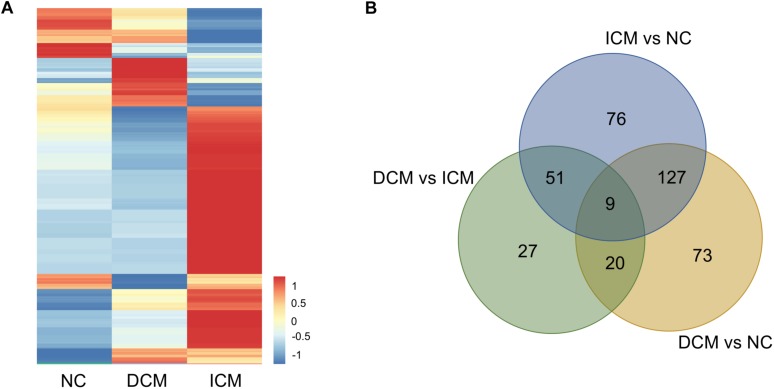
Proteomics analysis of the differentially expressed proteins. **(A)** Heatmap of the 286 differentially expressed proteins. **(B)** The amounts of common and unique differentially expressed proteins between groups were presented by Venn diagram. NC, control group; DCM, dilated cardiomyopathy; ICM, ischemic cardiomyopathy.

Proteomic analysis identified a total of 1,638 proteins that were subsequently used for comparative analysis. Among them, 286 proteins were differently expressed (Figure 2A). The list of these differentially expressed proteins were presented in Supplementary Material. We performed proteomics analysis between groups to identify differentially expressed proteins (Table 2). A total of 107 proteins were differentially expressed between the ICM and DCM groups (Figure 2B), which was lower compared to the proteins that were differentially expressed between ICM, DCM and the normal heart. This suggests that the pathophysiological changes of heart failure induced by ICM and DCM were only partially similar.

**TABLE 2:** The amounts of differentially expressed proteins between groups.

**Groups**	**Down-regulated**	**Up-regulated**	**Total**
DCM vs. NC	95	134	229
ICM vs. NC	108	155	263
ICM vs. DCM	38	69	107

*DCM, dilated cardiomyopathy; ICM, ischemic cardiomyopathy; NC, control group.*

### GO and KEGG Pathway Analysis

**FIGURE 3:**
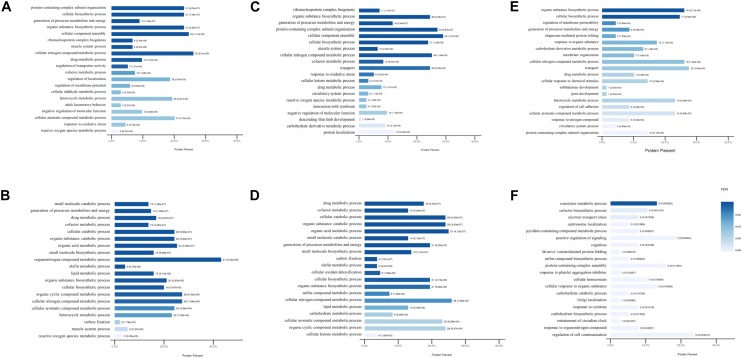
The modulated Gene Ontology biological processes terms. **(A)** Up and **(B)** down regulated processes in DCM group vs. NC group. **(C)** Up and **(D)** down regulated processes in ICM group vs. NC group. **(E)** Up and **(F)** down regulated processes in ICM group vs. DCM group.

To further assess the potential significance of the differentially expressed proteins to biological functions, GO and KEGG pathway analysis were performed by calculating the enrichment according to the up- and down-regulated proteins separately. The modulated GO biological process terms between the groups are presented in Figure 3. Compared to mice in the control group, the up-regulated proteins were modulated in protein-containing complex subunit organization, cellular biosynthetic process, organic substance biosynthetic process, and ribonucleoprotein complex biogenesis processes in DCM and ICM mouse hearts. The down-regulated proteins were modulated in multiple metabolic and muscle system processes. Furthermore, biological processes including biosynthetic, metabolic, membrane organization, protein folding were up-regulated in ICM hearts compared with DCM hearts. Electron transport chain, centrosome localization, some other biosynthetic and metabolic processes were down-regulated. Although the biological process modulation was similar between the two groups, the differentially expressed proteins modulated between the DCM and ICM hearts were different.

**FIGURE 4:**
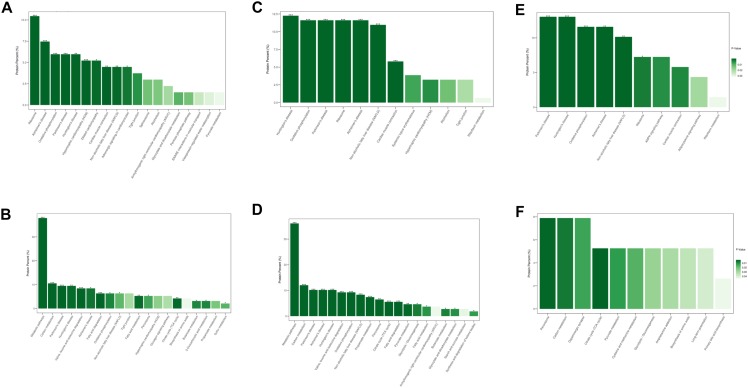
The modulated KEGG pathways according to the differentially expressed proteins between groups. **(A)** Up and **(B)** down regulated pathways in DCM group vs. NC group. **(C)** Up and **(D)** down regulated pathways in ICM group vs. NC group. **(E)** Up and **(F)** down regulated pathways in ICM group vs. DCM group. ^*^P < 0.05, ^∗∗^*P* < 0.01, ^∗∗∗^*P* < 0.001.

Similar results were observed after KEGG pathway analysis (Figure 4). Compared to normal control hearts, the up-regulated proteins were modulated in cardiomyopathy pathways, tight junction, ribosome and metabolism related diseases pathways in both DCM and ICM hearts. While the down-regulated proteins were modulated in oxidative phosphorylation, citrate cycle, and metabolism pathways including carbon, fat acid, amino acid metabolism. We further identified the different pathophysiological changes that arose in DCM and ICM hearts. Except for metabolism and oxidative phosphorylation pathways, the up-regulated proteins of ICM hearts were also modulated in the AMPK signaling and adipocytokine pathways.

### Network Analysis Using IPA

**FIGURE 5:**
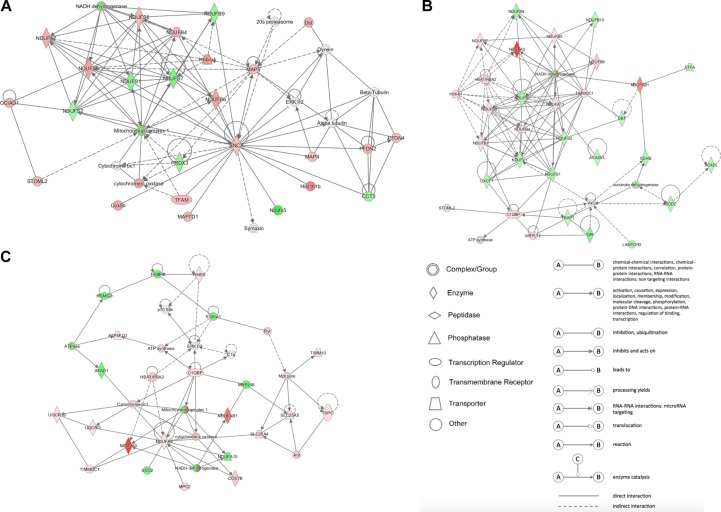
The network patterns built using IPA based on the differentially expressed proteins related to metabolism between **(A)** DCM and NC groups, **(B)** ICM and NC groups, and **(C)** DCM and ICM hearts. Red, up-regulation; green, down-regulation.

IPA analysis was used to build networks for the differentially expressed proteins. Metabolism related biology processes were significantly altered in DCM and ICM hearts. Hence, metabolism related proteins were selected to construct networks based on protein-protein interaction between the groups. The constructed networks are presented in Figure 5. The networks revealed different metabolic altered patterns in DCM and ICM hearts which indicated heterogeneity of heart failure induced by different factors.

### Western Blot Validation

**FIGURE 6:**
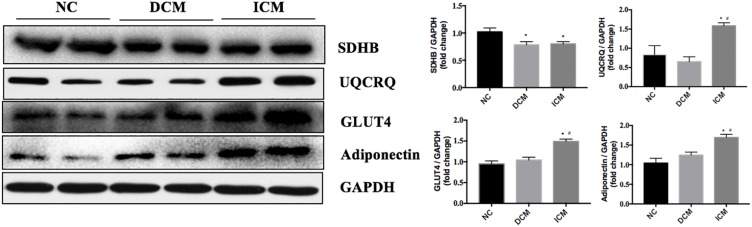
Validation of four differentially expressed proteins by Western blotting. GAPDH was used as the internal control. *n* = 4 per group. ^*^P < 0.05 vs. NC group, ^#^*P* < 0.05 vs. DCM group. NC, control group; DCM, dilated cardiomyopathy; ICM, ischemic cardiomyopathy.

To validate our proteomic results, four proteins related to metabolism and AMPK pathways were selected for confirmation by Western Blot analysis. Based on the proteomic results, SDHB protein expression was down-regulated in both ICM and DCM hearts, while UQCRQ, GLUT4 and adiponectin were up-regulated in only the ICM heart. Western blot results were consistent with the proteomics results and are presented in Figure 6.

### AMPK Activation and ATP Production in HF

**FIGURE 7:**
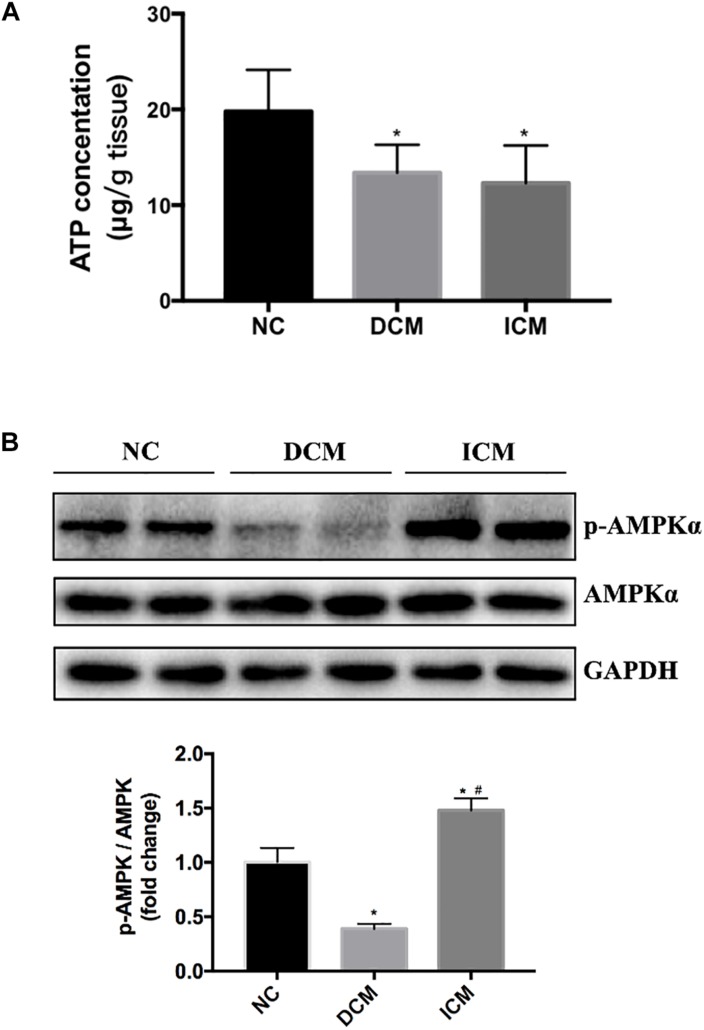
**(A)** ATP concentration significantly decreased in both DCM and ICM hearts. **(B)** Protein expression levels of phospho-AMPKα and total-AMPKα were determined by Western blotting. *n* = 4 per group. ^*^P < 0.05 vs. NC group, ^#^*P* < 0.05 vs. DCM group. NC, control group; DCM, dilated cardiomyopathy; ICM, ischemic cardiomyopathy.

Bioinformatics analysis suggested that oxidative phosphorylation pathway was disturbed in both ICM and DCM hearts. Furthermore, AMPK signaling pathway is differentially expressed between ICM and DCM. Thus, ATP concentration in failing and normal hearts were measured. ATP concentration significantly decreased in both DCM and ICM hearts (Figure 7A). Protein expression of phospho-AMPKα and total-AMPKα were also investigated by Western blot. Interestingly, Western blot demonstrated different status of AMPKα phosphorylation in DCM and ICM hearts. The protein expression of phospho-AMPKα decreased significantly in DCM, but increased in ICM (Figure 7B).

## Discussion

In the present study, a proteomic analysis was performed in mouse heart tissues after ICM and DCM to investigate cardiac protein expression changes in HF. The study was performed to decipher the molecular mechanisms related to the development of HF. Previous studies have reported protein expression changes in DCM (Nishtala et al., 2011) or ICM (Xiang et al., 2011) hearts separately using murine models. However, to the best of our knowledge, there have been no proteomic analysis performed to directly compare DCM and ICM hearts in murine HF models. In this study, we emphasized the different molecular mechanisms in HF induced by different pathophysiologic factors.

Previous studies have compared the proteome in ICM and DCM hearts in human patients and demonstrated the different protein expression profiles (Wei et al., 2009; Roselló-Lletí et al., 2012; Lionetti et al., 2014). Similarly, several proteins in mitochondria respiratory chain, cardiac metabolism pathways and stress response were significantly modulated in failing hearts and differentially expressed between ICM and DCM (Roselló-Lletí et al., 2012; Lionetti et al., 2014). These results were in keeping with our findings. However, DCM in human patients can be caused by different etiologies including genic mutation, viral infection, cardiotoxic agents and in many cases the cause remains unclear (Weintraub et al., 2017). Proteome studies in human hearts can hardly show the precise pathophysiologic mechanism of a specific etiology. Thus, we perform this study using animal models with better homogeneity so as to offer more precise information for further human studies.

ATP concentration was decreased in both DCM and ICM heart in our study. It is well-known that the heart has extremely high metabolic activity, requiring high ATP production via oxidative phosphorylation (Doenst et al., 2013; Fillmore et al., 2014). Myocardial ATP levels have been demonstrated to be decreased by 20–30% in HF patients and inadequate production of ATP induced impaired contractile function (Taha and Lopaschuk, 2007). Mitochondria is the key organelle for oxidative phosphorylation (Genova and Lenaz, 2014; Santulli et al., 2015; Brand, 2016). Several proteins in oxidative phosphorylation pathway significantly changed in failing hearts and indeed caused ATP production disturbance.

Proteomic analysis and western blot analysis demonstrated increased UQCRQ expression in ICM hearts, but not in DCM hearts. UQCRQ is a subunit of cytochrome c reductase complex III and is a part of the oxidative phosphorylation pathway and mitochondrial respiratory chain. Cytochrome c reductase complex III, including UQCRQ, UQCRC1, UQCRC2, are oxidatively damaged in response to infection in chagasic myocarditis hearts (Wen and Garg, 2004). Mutations in UQCRQ results in mitochondrial complex III deficiency and are usually presented as an autosomal-recessive nonlethal phenotype for severe psychomotor retardation and extrapyramidal symptoms (Barel et al., 2008). UQCRQ is differentially expressed in venous thromboembolism patients and may be a potential biomarker (Zhou et al., 2015). Cytochrome c reductase complex III has been identified as the main producer of superoxide and derived reactive oxygen species (ROS) within the mitochondrial respiratory chain (Dröse and Brandt, 2012; Bleier and Dröse, 2013). Ischemia-reperfusion initiates oxidative damage, cell death and aberrant immune responses through the production of mitochondrial ROS in the heart (Chouchani et al., 2014). Thus, UQCRQ may be a bridge between mitochondrial ROS production and myocardial dysfunction in ICM hearts.

GO enrichment and KEGG pathway analysis suggested that the differentially expressed proteins were mainly modulated in metabolic processes in both DCM and ICM hearts. The differentially expressed proteins between the two groups were also modulated in metabolic processes. Disturbance in metabolic process is an important initiator for heart failure (Ingwall, 2009; Ardehali et al., 2012). AMPK has been regarded as an important metabolic regulator in the heart (Arad et al., 2007; Zaha and Young, 2012). AMPK acts as a sensor of cellular ATP level and will be activated by phosphorylation when ATP is depleted. It has been demonstrated that AMPK is rapidly activated during myocardial ischemia (Dyck and Lopaschuk, 2006) which is also identified in this study. Activated AMPK promotes both fatty acid oxidation and glucose uptake and glycolysis which is important to the metabolic adaptation to ischemia (Zaha and Young, 2012). GLUT4 is the major transporter responsible for glucose uptake across the sarcolemma in cardiomyocytes (Shao and Tian, 2015). It has been reported that ischemia could result in increased myocardial glucose utilization and glucose transport activity due to the translocation of GLUT4 to the sarcolemma mediated via AMPK (Russell et al., 1999). Additionally, GLUT4 is highly localized in the ischemic region, but not in the non-ischemic regions (Young et al., 2000). GLUT4 knock-out mice display a seriously impaired response to ischemia (Dutka et al., 2006). The increased GLUT4 expression and glucose uptake and metabolism in the ischemic myocardium may be a protective response to prevent ischemic cardiomyocytes from irreparable damage.

Adiponectin has been shown to play an important role in cardioprotective properties (Matsuzawa et al., 2004; Tao et al., 2007; Takano et al., 2009; Shibata et al., 2012). Furthermore, adiponectin protects against cardiac ischemia-reperfusion injury via activating AMPK (Shibata et al., 2005). Previous studies have demonstrated that, besides adipocytes, adiponectin can also be synthesized and secreted by several other cells, including endothelial cells, macrophages as well as cardiomyocytes (Piñeiro et al., 2005; Fujioka et al., 2006). Locally secreted adiponectin may play a more direct role in the heart. In MI mouse models, overexpression of adiponectin in the heart results in decreased LV dilatation and improved LV function (Shibata et al., 2007). While in adiponectin KO mice, myocardial apoptosis and infarct size were significantly enhanced accompanied by increased nitric oxide, superoxide, and their cytotoxic reaction products (Tao et al., 2007). The increased adiponectin expression in ICM hearts may serve a protective role in the ischemic areas of the myocardial tissue. Nevertheless, up-regulated protein expression of GLUT4, adiponectin and phospho-AMPK was not detected in CVB3 induced DCM heart. Previous studies have demonstrated that AMPK activation alleviates cardiac fibroblast proliferation (Jiang et al., 2016) and restricts virus replication by inhibiting lipid accumulation *in vitro* (Xie et al., 2015). However, few studies have been made to investigating AMPK activation in CVB3 induced DCM models. Suppression of AMPK activation may be a critical mechanism in DCM development induced by CVB3 infection which can be investigate further.

In summary, oxidative phosphorylation, cardiac metabolism and protein folding play critical roles in the pathogenesis of heart failure. Diverse changes in protein expression profiles were observed in failing hearts induced by either MI or CVB3 infection. This demonstrates the heterogeneity of heart failure. Understanding the differences in proteome profiles during HF could offer more precise therapeutic options for patients.

## Data Availability

All datasets for this study are included in the manuscript and the Supplementary Files.

## Ethics Statement

This study was carried out in accordance with the Guide for the Care and Use of Laboratory Animals published by the US National Institutes of Health (NIH Publication No. 85-23, revised 1996). The protocol was approved by the Animal Care and Use Committee of Fudan University Zhongshan Hospital.

## Author Contributions

DL, YX, and ZC established the animal models, performed the proteomics analysis, and drafted the manuscript. AC, YW, and JJ performed the echocardiography, histology, and western blotting. AS and YZ revised the manuscript. JQ and JG designed the study.

## Conflict of Interest Statement

The authors declare that the research was conducted in the absence of any commercial or financial relationships that could be construed as a potential conflict of interest.

## Footnotes

1 https://CRAN.R-project.org/package=pheatmap

2 http://www.genome.jp/kegg

3 www.string-db.org
