# Supplementary material for: Cardiac Proteome Profiling in Ischemic and Dilated Cardiomyopathy Mouse Models
Source: Front Physiol. 2019 Jun 18;10:750. doi: 10.3389/fphys.2019.00750 (PMC6591471; doi:10.3389/fphys.2019.00750)
Supplement: Supplementary file 1 [file Table_1.pdf]

Supplementary Table S1. List of the differentially expressed proteins identified by proteomics. Fold change was calculated as a ratio of relative protein abundance. Protein expression was regarded as up-regulated when fold change > 1.5, and down-regulated when fold change < 0.67. (NC, control group; DCM, dilated cardiomyopathy; ICM, ischemic cardiomyopathy.)

Accession	Protein names	Protein annotations	NC	DCM			ICM				
			Relative protein abundance	Relative protein abundance	Fold change vs NC	Regulation vs NC	Relative protein abundance	Fold change vs NC	Regulation vs NC	Fold change vs DCM	Regulation vs DCM
P51667	MLRV	Myosin regulatory light chain 2, ventricular/cardiac muscle isoform (MLC-2) (MLC-2v) (Myosin light chain 2, slow skeletal/ventricular muscle isoform) (MLC-2s/v)	1020000	667000	0.65	Down	666000	0.65	Down	1.00	-
P23927	CRYAB	Alpha-crystallin B chain (Alpha(B)-crystallin) (P23)	395000	554000	1.40	-	681000	1.72	Up	1.23	-
F8WID5	F8WID5	Tropomyosin alpha-1 chain	17500	37000	2.11	Up	34600	1.98	Up	0.94	-
P02088	HBB1	Hemoglobin subunit beta-1 (Beta-1-globin) (Hemoglobin beta-1 chain) (Hemoglobin beta-major chain)	2740	6970	2.54	Up	6920	2.53	Up	0.99	-
Q9D3D9	ATPD	ATP synthase subunit delta, mitochondrial (ATP synthase F1 subunit delta) (F-ATPase delta subunit)	23500	25000	1.06	-	49200	2.09	Up	1.97	Up
Q9CR61	NDUB7	NADH dehydrogenase [ubiquinone] 1 beta subcomplex subunit 7 (Complex I-B18) (CI-B18) (NADH-ubiquinone oxidoreductase B18 subunit)	90800	124000	1.37	-	146000	1.61	Up	1.18	-
P21550	ENOB	Beta-enolase (EC 4.2.1.11) (2-phospho-D-glycerate hydro-lyase) (Enolase 3) (Muscle-specific enolase) (MSE) (Skeletal muscle enolase)	119000	136000	1.14	-	90600	0.76	-	0.67	Down
P01942	HBA	Hemoglobin subunit alpha (Alpha-globin) (Hemoglobin alpha chain)	44000	35000	0.80	-	137000	3.11	Up	3.91	Up
P07759	SPA3K	Serine protease inhibitor A3K (Serpin A3K) (Contrapsin) (SPI-2)	63200	106000	1.68	Up	103000	1.63	Up	0.97	-
P68134	ACTS	Actin, alpha skeletal muscle (Alpha-actin-1) [Cleaved	44700	34800	0.78	-	16700	0.37	Down	0.48	Down

		into: Actin, alpha skeletal muscle, intermediate form]								
A0A1D5RLD8	A0A1D5RLD8	Glyceraldehyde-3-phosphate dehydrogenase (EC 1.2.1.12)	17900	16400	0.92	-	34400	1.92	Up	2.10	Up
Q9R0Y5	KAD1	Adenylate kinase isoenzyme 1 (AK 1) (EC 2.7.4.3) (EC 2.7.4.6) (ATP-AMP transphosphorylase 1) (ATP:AMP phosphotransferase) (Adenylate monophosphate kinase) (Myokinase)	11400	9500	0.83	-	15100	1.32	-	1.59	Up
Q8K3J1	NDUS8	NADH dehydrogenase [ubiquinone] iron-sulfur protein 8, mitochondrial (EC 1.6.99.3) (EC 7.1.1.2) (Complex I-23kD) (CI-23kD) (NADH-ubiquinone oxidoreductase 23 kDa subunit)	531	1230	2.32	Up	1100	2.07	Up	0.89	-
Q60931	VDAC3	Voltage-dependent anion-selective channel protein 3 (VDAC-3) (mVDAC3) (Outer mitochondrial membrane protein porin 3)	10700	5420	0.51	Down	6620	0.62	Down	1.22	-
P10854	H2B1M	Histone H2B type 1-M (H2B 291B)	14900	27700	1.86	Up	39400	2.64	Up	1.42	-
P60710	ACTB	Actin, cytoplasmic 1 (Beta-actin) [Cleaved into: Actin, cytoplasmic 1, N-terminally processed]	2630	1300	0.49	Down	1040	0.40	Down	0.80	-
Q8BMS1	ECHA	Trifunctional enzyme subunit alpha, mitochondrial (TP-alpha) [Includes: Long-chain enoyl-CoA hydratase (EC 4.2.1.17); Long chain 3-hydroxyacyl-CoA dehydrogenase (EC 1.1.1.211)]	118000	58700	0.50	Down	71400	0.61	Down	1.22	-
P14602	HSPB1	Heat shock protein beta-1 (HspB1) (Growth-related 25 kDa protein) (Heat shock 25 kDa protein) (HSP 25) (Heat shock 27 kDa protein) (HSP 27) (p25)	51400	69000	1.34	-	107000	2.08	Up	1.55	Up
Q8R5L1	Q8R5L1	Complement component 1 Q subcomponent-binding protein, mitochondrial (Complement component 1, q subcomponent binding protein) (Complement component 1, q subcomponent binding protein, isoform CRA_b) (p32-RACK)	5000	7430	1.49	-	11900	2.38	Up	1.60	Up
Q9D0K2	SCOT1	Succinyl-CoA:3-ketoacid coenzyme A transferase 1,	34700	28600	0.82	-	22500	0.65	Down	0.79	-

		mitochondrial (EC 2.8.3.5) (3-oxoacid CoA-transferase 1) (Somatic-type succinyl-CoA:3-oxoacid CoA-transferase) (SCOT-s)									
Q99LC5	ETFA	Electron transfer flavoprotein subunit alpha, mitochondrial (Alpha-ETF)	118000	79900	0.68	-	67600	0.57	Down	0.85	-
Q8BUJ6	Q8BUJ6	Titin (Fragment)	99000	124000	1.25	-	165000	1.67	Up	1.33	-
Q9CZ19	Q9CZ19	Myosin light chain 4 (Myosin, light polypeptide 4)	6730	26800	3.98	Up	30800	4.58	Up	1.15	-
P20108	PRDX3	Thioredoxin-dependent peroxide reductase, mitochondrial (EC 1.11.1.15) (Antioxidant protein 1) (AOP-1) (PRX III) (Perioredoxin-3) (Protein MER5)	134000	77900	0.58	Down	56500	0.42	Down	0.73	-
P13541	MYH3	Myosin-3 (Myosin heavy chain 3)	166	274	1.65	Up	408	2.46	Up	1.49	Up
Q99KI0	ACON	Aconitate hydratase, mitochondrial (Aconitase) (EC 4.2.1.3) (Citrate hydro-lyase)	32700	17700	0.54	Down	19200	0.59	Down	1.08	-
P19123	TNNC1	Troponin C, slow skeletal and cardiac muscles (TN-C)	42900	24100	0.56	Down	24500	0.57	Down	1.02	-
P50544	ACADV	Very long-chain specific acyl-CoA dehydrogenase, mitochondrial (EC 1.3.8.9) (MVLCAD) (VLCAD)	122000	92700	0.76	-	79400	0.65	Down	0.86	-
P14211	CALR	Calreticulin (CRP55) (Calregulin) (Endoplasmic reticulum resident protein 60) (ERp60) (HACBP)	19100	28100	1.47	-	29900	1.57	Up	1.06	-
B1AR69	B1AR69	Myosin, heavy polypeptide 13, skeletal muscle	874	528	0.60	Down	317	0.36	Down	0.60	Down
P35385	HSPB7	Heat shock protein beta-7 (HspB7) (Cardiovascular heat shock protein) (cvHsp) (Heat shock protein 25 kDa 2) (Protein p19/6.8)	2610	2200	0.84	-	3760	1.44	-	1.71	Up
Q8CGP7	H2A1K	Histone H2A type 1-K	36300	153000	4.21	Up	102000	2.81	Up	0.67	Down
A0A0R4J083	A0A0R4J083	Long-chain-specific acyl-CoA dehydrogenase, mitochondrial	91100	66500	0.73	-	55700	0.61	Down	0.84	-
A0A0N4SVQ1	A0A0N4SVQ1	Cytochrome c oxidase subunit NDUFA4	215000	270000	1.26	-	510000	2.37	Up	1.89	Up
Q9ERS2	NDUAD	NADH dehydrogenase [ubiquinone] 1 alpha subcomplex subunit 13 (Cell death regulatory protein GRIM-19) (Complex I-B16.6) (CI-B16.6) (Gene	45200	64200	1.42	-	78700	1.74	Up	1.23	-

		associated with retinoic and interferon-induced mortality 19 protein) (GRIM-19) (Gene associated with retinoic and IFN-induced mortality 19 protein) (NADH-ubiquinone oxidoreductase B16.6 subunit)								
Q60605	MYL6	Myosin light polypeptide 6 (17 kDa myosin light chain) (LC17) (Myosin light chain 3) (MLC-3) (Myosin light chain alkali 3) (Myosin light chain A3) (Smooth muscle and nonmuscle myosin light chain alkali 6)	3990	7010	1.76	Up	8480	2.13	Up	1.21	-
P35486	ODPA	Pyruvate dehydrogenase E1 component subunit alpha, somatic form, mitochondrial (EC 1.2.4.1) (PDHE1-A type I)	24700	17800	0.72	-	15600	0.63	Down	0.88	-
G5E902	G5E902	MCG10343, isoform CRA_b (Phosphate carrier protein, mitochondrial)	20400	14700	0.72	-	54800	2.69	Up	3.73	Up
P54071	IDHP	Isocitrate dehydrogenase [NADP], mitochondrial (IDH) (EC 1.1.1.42) (ICD-M) (IDP) (NADP(+)-specific ICDH) (Oxalosuccinate decarboxylase)	244000	163000	0.67	Down	131000	0.54	Down	0.80	-
P11531	DMD	Dystrophin	19600	32400	1.65	Up	33600	1.71	Up	1.04	-
Q91WD5	NDUS2	NADH dehydrogenase [ubiquinone] iron-sulfur protein 2, mitochondrial (EC 1.6.99.3) (EC 7.1.1.2) (Complex I-49kD) (CI-49kD) (NADH-ubiquinone oxidoreductase 49 kDa subunit)	32200	20800	0.65	Down	20300	0.63	Down	0.98	-
P70670	NACAM	Nascent polypeptide-associated complex subunit alpha, muscle-specific form (Alpha-NAC, muscle-specific form) (skNAC)	16300	27500	1.69	Up	25200	1.55	Up	0.92	-
P48962	ADT1	ADP/ATP translocase 1 (ADP,ATP carrier protein 1) (ADP,ATP carrier protein, heart/skeletal muscle isoform T1) (Adenine nucleotide translocator 1) (ANT 1) (Solute carrier family 25 member 4) (mANC1)	55200	46900	0.85	-	122000	2.21	Up	2.60	Up
H3BJQ7	H3BJQ7	Peroxiredoxin-5, mitochondrial	36000	36600	1.02	-	24200	0.67	-	0.66	Down
Q9DCS9	NDUBA	NADH dehydrogenase [ubiquinone] 1 beta subcomplex	187000	126000	0.67	-	121000	0.65	Down	0.96	-

		subunit 10 (Complex I-PDSW) (CI-PDSW) (NADH-ubiquinone oxidoreductase PDSW subunit)								
P56565	S10A1	Protein S100-A1 (S-100 protein alpha chain) (S-100 protein subunit alpha) (S100 calcium-binding protein A1)	112000	36800	0.33	Down	25400	0.23	Down	0.69	-
Q8CI94	PYGB	Glycogen phosphorylase, brain form (EC 2.4.1.1)	30600	17400	0.57	Down	18000	0.59	Down	1.03	-
Q9Z2I9	SUCB1	Succinate--CoA ligase [ADP-forming] subunit beta, mitochondrial (EC 6.2.1.5) (ATP-specific succinyl-CoA synthetase subunit beta) (A-SCS) (Succinyl-CoA synthetase beta-A chain) (SCS-betaA)	41600	31300	0.75	-	26700	0.64	Down	0.85	-
P62082	RS7	40S ribosomal protein S7	36600	65800	1.80	Up	80500	2.20	Up	1.22	-
P09671	SODM	Superoxide dismutase [Mn], mitochondrial (EC 1.15.1.1)	11600	8410	0.73	-	7320	0.63	Down	0.87	-
P0DP28	CALM3	Calmodulin-3	6440	15100	2.34	Up	8600	1.34	-	0.57	Down
P06745	G6PI	Glucose-6-phosphate isomerase (GPI) (EC 5.3.1.9) (Autocrine motility factor) (AMF) (Neuroleukin) (NLK) (Phosphoglucose isomerase) (PGI) (Phosphohexose isomerase) (PHI)	7290	3050	0.42	Down	3080	0.42	Down	1.01	-
P51881	ADT2	ADP/ATP translocase 2 (ADP,ATP carrier protein 2) (Adenine nucleotide translocator 2) (ANT 2) (Solute carrier family 25 member 5) [Cleaved into: ADP/ATP translocase 2, N-terminally processed]	31400	19400	0.62	Down	38300	1.22	-	1.97	Up
B7FAV1	B7FAV1	Filamin, alpha (Filamin-A) (Fragment)	9200	11400	1.24	-	14000	1.52	Up	1.23	-
Q9QVP4	MLRA	Myosin regulatory light chain 2, atrial isoform (MLC-2a) (MLC2a) (Myosin light chain 2a) (Myosin regulatory light chain 7)	3930	21600	5.50	Up	57500	14.63	Up	2.66	Up
Q3UIU2	NDUB6	NADH dehydrogenase [ubiquinone] 1 beta subcomplex subunit 6 (Complex I-B17) (CI-B17) (NADH-ubiquinone oxidoreductase B17 subunit)	6330	14400	2.27	Up	20200	3.19	Up	1.40	-
Q9WTR5	CAD13	Cadherin-13 (Heart cadherin) (H-cadherin) (Truncated	5710	7070	1.24	-	9370	1.64	Up	1.33	-

		cadherin) (T-cad) (T-cadherin)								
P19536	COX5B	Cytochrome c oxidase subunit 5B, mitochondrial (Cytochrome c oxidase polypeptide Vb)	2760	6170	2.24	Up	6140	2.22	Up	1.00	-
P31786	ACBP	Acyl-CoA-binding protein (ACBP) (Diazepam-binding inhibitor) (DBI) (Endozepine) (EP)	1950	3260	1.67	Up	1630	0.84	-	0.50	Down
A2AQP0	MYH7B	Myosin-7B (Myosin cardiac muscle beta chain) (Myosin heavy chain 7B, cardiac muscle beta isoform)	1130	1570	1.39	-	1990	1.76	Up	1.27	-
P62806	H4	Histone H4	18700	19700	1.05	-	32900	1.76	Up	1.67	Up
Q91VD9	NDUS1	NADH-ubiquinone oxidoreductase 75 kDa subunit, mitochondrial (EC 1.6.99.3) (EC 7.1.1.2) (Complex I-75kD) (CI-75kD)	18600	9560	0.51	Down	10200	0.55	Down	1.07	-
P63242	IF5A1	Eukaryotic translation initiation factor 5A-1 (eIF-5A-1) (eIF-5A1) (Eukaryotic initiation factor 5A isoform 1) (eIF-5A) (eIF-4D)	1900	1590	0.84	-	7770	4.09	Up	4.89	Up
Q8BMF4	ODP2	Dihydrolipoyllysine-residue acetyltransferase component of pyruvate dehydrogenase complex, mitochondrial (EC 2.3.1.12) (Dihydrolipoamide acetyltransferase component of pyruvate dehydrogenase complex) (Pyruvate dehydrogenase complex component E2) (PDC-E2) (PDCE2)	103000	102000	0.99	-	64200	0.62	Down	0.63	Down
Q9CQH3	NDUB5	NADH dehydrogenase [ubiquinone] 1 beta subcomplex subunit 5, mitochondrial (Complex I-SGDH) (CI-SGDH) (NADH-ubiquinone oxidoreductase SGDH subunit)	96300	137000	1.42	-	191000	1.98	Up	1.39	-
J3QP71	J3QP71	Basigin (Fragment)	35200	28800	0.82	-	19700	0.56	Down	0.68	-
O88569	ROA2	Heterogeneous nuclear ribonucleoproteins A2/B1 (hnRNP A2/B1)	12000	19500	1.63	Up	25300	2.11	Up	1.30	-
Q9JHI5	IVD	Isovaleryl-CoA dehydrogenase, mitochondrial (IVD) (EC 1.3.8.4)	14500	12100	0.83	-	8580	0.59	Down	0.71	-
A2A838	A2A838	Protein 4.1 (Fragment)	422	653	1.55	Up	649	1.54	Up	0.99	-

Q80XN0	BDH	D-beta-hydroxybutyrate dehydrogenase, mitochondrial (EC 1.1.1.30) (3-hydroxybutyrate dehydrogenase) (BDH)	17100	9910	0.58	Down	11200	0.65	Down	1.13	-
E9Q401	RYR2	Ryanodine receptor 2 (RYR-2) (RyR2) (Cardiac muscle ryanodine receptor) (Cardiac muscle ryanodine receptor-calcium release channel) (Type 2 ryanodine receptor)	4070	2570	0.63	Down	4200	1.03	-	1.63	Up
P19324	SERPH	Serpin H1 (47 kDa heat shock protein) (Collagen-binding protein) (Colligin) (Serine protease inhibitor J6)	19900	31200	1.57	Up	33600	1.69	Up	1.08	-
Q99N15	Q99N15	17beta-hydroxysteroid dehydrogenase type 10/short chain L-3-hydroxyacyl-CoA dehydrogenase (3-hydroxyacyl-CoA dehydrogenase type-2) (Hydroxysteroid (17-beta) dehydrogenase 10)	22000	13300	0.60	Down	11300	0.51	Down	0.85	-
Q9JHJ0	TMOD3	Tropomodulin-3 (Ubiquitous tropomodulin) (U-Tmod)	12600	20200	1.60	Up	19700	1.56	Up	0.98	-
P10922	H10	Histone H1.0 (Histone H1') (Histone H1(0)) (MyD196) [Cleaved into: Histone H1.0, N-terminally processed]	4410	19900	4.51	Up	52300	11.86	Up	2.63	Up
P97443	SMYD1	Histone-lysine N-methyltransferase Smyd1 (EC 2.1.1.43) (CD8b-opposite) (SET and MYND domain-containing protein 1) (Zinc finger protein BOP) (m-BOP)	13700	15300	1.12	-	8660	0.63	Down	0.57	Down
E9PV24	FIBA	Fibrinogen alpha chain [Cleaved into: Fibrinopeptide A; Fibrinogen alpha chain]	38300	65100	1.70	Up	42400	1.11	-	0.65	Down
A0A0G2JDZ4	A0A0G2JDZ4	Myosin regulatory light chain 10	385	849	2.20	Up	607	1.58	Up	0.71	-
P14733	LMNB1	Lamin-B1	21500	23800	1.11	-	15500	0.72	-	0.65	Down
O35887	CALU	Calumenin (Crocalbin)	3810	8190	2.15	Up	7730	2.03	Up	0.94	-
Q9WV35	ABEC2	C->U-editing enzyme APOBEC-2 (EC 3.5.4.36) (mRNA(cytosine(6666)) deaminase 2)	11000	4950	0.45	Down	4500	0.41	Down	0.91	-
Q9CRB8	MTFP1	Mitochondrial fission process protein 1 (Mitochondrial	41400	32800	0.79	-	50700	1.22	-	1.55	Up

		18 kDa protein) (MTP18)								
P51125	ICAL	Calpastatin (Calpain inhibitor)	12000	25100	2.09	Up	19100	1.59	Up	0.76	-
P62631	EF1A2	Elongation factor 1-alpha 2 (EF-1-alpha-2) (Eukaryotic elongation factor 1 A-2) (eEF1A-2) (Statin-S1)	7600	2500	0.33	Down	3200	0.42	Down	1.28	-
A2BE93	A2BE93	Protein SET (Fragment)	13900	27700	1.99	Up	23400	1.68	Up	0.84	-
Q9CQC7	NDUB4	NADH dehydrogenase [ubiquinone] 1 beta subcomplex subunit 4 (Complex I-B15) (CI-B15) (NADH-ubiquinone oxidoreductase B15 subunit)	9960	19500	1.96	Up	27500	2.76	Up	1.41	-
Q9EQ20	MMSA	Methylmalonate-semialdehyde dehydrogenase [acylating], mitochondrial (MMSDH) (Malonate-semialdehyde dehydrogenase [acylating]) (EC 1.2.1.18) (EC 1.2.1.27) (Aldehyde dehydrogenase family 6 member A1)	13200	8600	0.65	Down	8460	0.64	Down	0.98	-
A0A0R4J1E2	A0A0R4J1E2	Elongation factor 1-delta	589	770	1.31	-	908	1.54	Up	1.18	-
Q9CQ69	QCR8	Cytochrome b-c1 complex subunit 8 (Complex III subunit 8) (Complex III subunit VIII) (Ubiquinol-cytochrome c reductase complex 9.5 kDa protein) (Ubiquinol-cytochrome c reductase complex ubiquinone-binding protein QP-C)	30300	32100	1.06	-	56100	1.85	Up	1.75	Up
P09405	NUCL	Nucleolin (Protein C23)	15300	26500	1.73	Up	23500	1.54	Up	0.89	-
P47934	CACP	Carnitine O-acetyltransferase (Carnitine acetylase) (EC 2.3.1.7) (Carnitine acetyltransferase) (CAT) (CrAT)	25300	14800	0.58	Down	13700	0.54	Down	0.93	-
Q06185	ATP5I	ATP synthase subunit e, mitochondrial (ATPase subunit e) (ATP synthase membrane subunit e)	6990	17500	2.50	Up	27800	3.98	Up	1.59	Up
Q924X2	CPT1B	Carnitine O-palmitoyltransferase 1, muscle isoform (CPT1-M) (EC 2.3.1.21) (Carnitine O-palmitoyltransferase I, muscle isoform) (CPT I) (CPTI-M) (Carnitine palmitoyltransferase 1B)	20000	10000	0.50	Down	18700	0.94	-	1.87	Up
Q99PT1	GDIR1	Rho GDP-dissociation inhibitor 1 (Rho GDI 1) (GDI-1)	4760	11000	2.31	Up	12700	2.67	Up	1.15	-

		(Rho-GDI alpha)									
P61027	RAB10	Ras-related protein Rab-10	2410	1850	0.77	-	3250	1.35	-	1.76	Up
Q7TMG8	Q7TMG8	Glioblastoma amplified sequence (Protein NipSnap homolog 2)	11500	3920	0.34	Down	4100	0.36	Down	1.05	-
P47963	RL13	60S ribosomal protein L13 (A52)	2370	4230	1.78	Up	5090	2.15	Up	1.20	-
Q8CHT0	AL4A1	Delta-1-pyrroline-5-carboxylate dehydrogenase, mitochondrial (P5C dehydrogenase) (EC 1.2.1.88) (Aldehyde dehydrogenase family 4 member A1) (L-glutamate gamma-semialdehyde dehydrogenase)	5850	3830	0.65	Down	6350	1.09	-	1.66	Up
P62849	RS24	40S ribosomal protein S24	10300	18200	1.77	Up	34600	3.36	Up	1.90	Up
Q99MR8	MCCA	Methylcrotonoyl-CoA carboxylase subunit alpha, mitochondrial (MCCase subunit alpha) (EC 6.4.1.4) (3-methylcrotonyl-CoA carboxylase 1) (3-methylcrotonyl-CoA carboxylase biotin-containing subunit) (3-methylcrotonyl-CoA:carbon dioxide ligase subunit alpha)	7680	4040	0.53	Down	6400	0.83	-	1.58	Up
	RUXG	Small nuclear ribonucleoprotein G (snRNP-G) (Sm protein G) (Sm-G) (SmG)	1950	3140	1.61	Up	3930	2.02	Up	1.25	-
P62073	TIM10	Mitochondrial import inner membrane translocase subunit Tim10	4900	7160	1.46	-	11100	2.27	Up	1.55	Up
P68433	H31	Histone H3.1	8250	58000	7.03	Up	86100	10.44	Up	1.48	-
P08032	SPTA1	Spectrin alpha chain, erythrocytic 1 (Erythroid alpha-spectrin)	755	526	0.70	-	880	1.17	-	1.67	Up
P19001	K1C19	Keratin, type I cytoskeletal 19 (Cytokeratin-19) (CK-19) (Keratin-19) (K19)	1500	2920	1.95	Up	2640	1.76	Up	0.90	-
Q923D2	BLVRB	Flavin reductase (NADPH) (FR) (EC 1.5.1.30) (Biliverdin reductase B) (BVR-B) (EC 1.3.1.24) (Biliverdin-IX beta-reductase) (NADPH-dependent diaphorase) (NADPH-flavin reductase) (FLR)	833	873	1.05	-	1330	1.60	Up	1.52	Up
A0A1W2	A0A1W2	Ubiquitin-conjugating enzyme E2 N	13100	8510	0.65	Down	8460	0.65	Down	0.99	-

P7Z3	P7Z3										
P62843	RS15	40S ribosomal protein S15 (RIG protein)	233	364	1.56	Up	370	1.59	Up	1.02	-
Q6ZWN5	RS9	40S ribosomal protein S9	7050	11900	1.69	Up	14300	2.03	Up	1.20	-
D3Z1U9	D3Z1U9	NADH dehydrogenase [ubiquinone] flavoprotein 1, mitochondrial (EC 1.6.99.3) (EC 7.1.1.2)	8730	6140	0.70	-	5500	0.63	Down	0.90	-
A0A1B0GX27	A0A1B0GX27	Branched-chain-amino-acid aminotransferase (EC 2.6.1.42)	3190	3920	1.23	-	5100	1.60	Up	1.30	-
Q9CR21	ACPM	Acyl carrier protein, mitochondrial (ACP) (CI-SDAP) (NADH-ubiquinone oxidoreductase 9.6 kDa subunit)	6490	5260	0.81	-	49800	7.67	Up	9.47	Up
Q91WS0	CISD1	CDGSH iron-sulfur domain-containing protein 1 (MitoNEET)	2300	5370	2.33	Up	5770	2.51	Up	1.07	-
A1BN54	A1BN54	Alpha actinin 1a (Alpha-actinin-1)	1150	856	0.74	-	730	0.63	Down	0.85	-
Q3TCD4	Q3TCD4	Enoyl-CoA delta isomerase 2, mitochondrial	1010	812	0.80	-	666	0.66	Down	0.82	-
P42125	ECI1	Enoyl-CoA delta isomerase 1, mitochondrial (EC 5.3.3.8) (3,2-trans-enoyl-CoA isomerase) (Delta(3),Delta(2)-enoyl-CoA isomerase) (D3,D2-enoyl-CoA isomerase) (Dodecenoyl-CoA isomerase)	23700	12800	0.54	Down	10800	0.46	Down	0.84	-
Q9CPQ1	COX6C	Cytochrome c oxidase subunit 6C (Cytochrome c oxidase polypeptide VIc)	8820	9670	1.10	-	13900	1.58	Up	1.44	-
E9PZF0	E9PZF0	Nucleoside diphosphate kinase (EC 2.7.4.6)	14300	7650	0.53	Down	7470	0.52	Down	0.98	-
Q99J99	THTM	3-mercaptopyruvate sulfurtransferase (MST) (EC 2.8.1.2)	9110	4510	0.50	Down	7040	0.77	-	1.56	Up
P05125	ANF	Natriuretic peptides A (Prepronatriodilatin) [Cleaved into: Atrial natriuretic factor (ANF) (Atrial natriuretic peptide) (ANP); Auriculin-B; Auriculin-A; Atriopeptin-1 (Atriopeptin I); Atriopeptin-2 (Atriopeptin II)]	4090	10100	2.47	Up	8530	2.09	Up	0.84	-
B1ARA5	B1ARA5	60S ribosomal protein L26	4400	6460	1.47	-	13300	3.02	Up	2.06	Up
P56375	ACYP2	Acylphosphatase-2 (EC 3.6.1.7) (Acylphosphatase, muscle type isozyme) (Acylphosphate phosphohydrolase 2)	10600	26000	2.45	Up	25500	2.41	Up	0.98	-

P15626	GSTM2	Glutathione S-transferase Mu 2 (EC 2.5.1.18) (GST 5-5) (GST class-mu 2) (Glutathione S-transferase pmGT2)	8990	13400	1.49	Up	15500	1.72	Up	1.16	-
Q9DC70	NDUS7	NADH dehydrogenase [ubiquinone] iron-sulfur protein 7, mitochondrial (EC 1.6.99.3) (EC 7.1.1.2) (Complex I-20kD) (CI-20kD) (NADH-ubiquinone oxidoreductase 20 kDa subunit)	93500	43900	0.47	Down	50600	0.54	Down	1.15	-
Q99PR8	HSPB2	Heat shock protein beta-2 (HspB2)	4110	5220	1.27	-	6240	1.52	Up	1.20	-
P56392	CX7A1	Cytochrome c oxidase subunit 7A1, mitochondrial (Cytochrome c oxidase subunit VIIa-heart) (Cytochrome c oxidase subunit VIIa-H) (Cytochrome c oxidase subunit VIIa-muscle) (Cytochrome c oxidase subunit VIIa-M)	54300	72300	1.33	-	109000	2.01	Up	1.51	Up
P06151	LDHA	L-lactate dehydrogenase A chain (LDH-A) (EC 1.1.1.27) (LDH muscle subunit) (LDH-M)	15500	8910	0.57	Down	10100	0.65	Down	1.13	-
Q99LC3	NDUAA	NADH dehydrogenase [ubiquinone] 1 alpha subcomplex subunit 10, mitochondrial (Complex I-42kD) (CI-42kD) (NADH-ubiquinone oxidoreductase 42 kDa subunit)	8620	11600	1.35	-	7740	0.90	-	0.67	Down
P11983	TCPA	T-complex protein 1 subunit alpha (TCP-1-alpha) (CCT-alpha) (Tailless complex polypeptide 1A) (TCP-1-A) (Tailless complex polypeptide 1B) (TCP-1-B)	10000	10800	1.08	-	17900	1.79	Up	1.66	Up
P01902	HA1D	H-2 class I histocompatibility antigen, K-D alpha chain (H-2K(D))	7290	7080	0.97	-	15800	2.17	Up	2.23	Up
P10493	NID1	Nidogen-1 (NID-1) (Entactin)	4480	4880	1.09	-	8160	1.82	Up	1.67	Up
P53395	ODB2	Lipoamide acyltransferase component of branched-chain alpha-keto acid dehydrogenase complex, mitochondrial (EC 2.3.1.168) (Branched-chain alpha-keto acid dehydrogenase complex component E2) (BCKAD-E2) (BCKADE2) (Dihydrolipoamide	39300	26800	0.68	-	22800	0.58	Down	0.85	-

		acetyltransferase component of branched-chain alpha-keto acid dehydrogenase complex) (Dihydrolipoamide branched chain transacylase) (Dihydrolipoyllysine-residue (2-methylpropanoyl)transferase)									
Q99JB2	STML2	Stomatin-like protein 2, mitochondrial (SLP-2) (mslp2)	1780	3100	1.74	Up	2920	1.64	Up	0.94	-
P61982	1433G	14-3-3 protein gamma [Cleaved into: 14-3-3 protein gamma, N-terminally processed]	3900	2020	0.52	Down	1940	0.50	Down	0.96	-
Q9Z2D6	MECP2	Methyl-CpG-binding protein 2 (MeCp-2 protein) (MeCp2)	2770	5470	1.97	Up	5320	1.92	Up	0.97	-
P29595	NEDD8	NEDD8 (Neddylin) (Neural precursor cell expressed developmentally down-regulated protein 8) (NEDD-8) (Ubiquitin-like protein Nedd8)	31400	52500	1.67	Up	52200	1.66	Up	0.99	-
F8VPN4	F8VPN4	Amylo-1,6-glucosidase, 4-alpha-glucanotransferase	1700	1090	0.64	Down	1040	0.61	Down	0.95	-
Q8CBM2	Q8CBM2	Aspartyl/asparaginyl beta-hydroxylase	11100	14700	1.32	-	30800	2.77	Up	2.10	Up
Q9CYT6	CAP2	Adenylyl cyclase-associated protein 2 (CAP 2)	9480	11300	1.19	-	5140	0.54	Down	0.45	Down
Q8C845	Q8C845	EF-hand domain-containing protein D2 (Efhd2 protein)	6170	16200	2.63	Up	12400	2.01	Up	0.77	-
Q9D1D4	TMEDA	Transmembrane emp24 domain-containing protein 10 (21 kDa transmembrane-trafficking protein) (Transmembrane protein Tmp21) (p24 family protein delta-1) (p24delta1)	10600	12900	1.22	-	22500	2.12	Up	1.74	Up
P40630	TFAM	Transcription factor A, mitochondrial (mtTFA) (Testis-specific high mobility group protein) (TS-HMG)	9470	16400	1.73	Up	15700	1.66	Up	0.96	-
Q9R229	BMP10	Bone morphogenetic protein 10 (BMP-10)	2940	13700	4.66	Up	9030	3.07	Up	0.66	Down
Q68FD5	CLH1	Clathrin heavy chain 1	5680	7630	1.34	-	4470	0.79	-	0.59	Down
E9Q9C4	E9Q9C4	Actin-binding LIM protein 1	3020	5730	1.90	Up	5210	1.73	Up	0.91	-
Q99KV1	DJB11	DnaJ homolog subfamily B member 11 (APOBEC1-binding protein 2) (ABBP-2) (ER-associated DNAJ) (ER-associated Hsp40 co-chaperone) (Endoplasmic reticulum DNA J domain-containing protein 3) (ER-resident protein ERdj3) (ERdj3) (ERj3p)	6320	10700	1.69	Up	12900	2.04	Up	1.21	-

Q8BUY5	TIDC1	Complex I assembly factor TIMMDC1, mitochondrial (Translocase of inner mitochondrial membrane domain-containing protein 1) (TIMM domain containing-protein 1)	6380	5310	0.83	-	11400	1.79	Up	2.15	Up
Q9QZM0	UBQL2	Ubiquilin-2 (Chap1) (DSK2 homolog) (Protein linking IAP with cytoskeleton 2) (PLIC-2) (Ubiquitin-like product Chap1/Dsk2)	2990	2000	0.67	Down	1970	0.66	Down	0.99	-
P17665	COX7C	Cytochrome c oxidase subunit 7C, mitochondrial (Cytochrome c oxidase polypeptide VIIc)	486	658	1.36	-	782	1.61	Up	1.19	-
A0A087 WSN6	A0A087 WSN6	Fibronectin (Fibronectin 1, isoform CRA_c)	2480	3310	1.33	-	4150	1.67	Up	1.25	-
Q99NB1	ACS2L	Acetyl-coenzyme A synthetase 2-like, mitochondrial (EC 6.2.1.1) (Acetate--CoA ligase 2) (Acetyl-CoA synthetase 2) (AceCS2) (Acyl-CoA synthetase short-chain family member 1)	8510	6400	0.75	-	4830	0.57	Down	0.75	-
Q9DCV7	K2C7	Keratin, type II cytoskeletal 7 (Cytokeratin-7) (CK-7) (Keratin-7) (K7) (Type-II keratin Kb7)	1140	2640	2.32	Up	1880	1.65	Up	0.71	-
Q61646	HPT	Haptoglobin [Cleaved into: Haptoglobin alpha chain; Haptoglobin beta chain]	3590	13800	3.84	Up	13400	3.73	Up	0.97	-
P08551	NFL	Neurofilament light polypeptide (NF-L) (68 kDa neurofilament protein) (Neurofilament triplet L protein)	2010	1730	0.86	-	2600	1.29	-	1.50	Up
G5E850	G5E850	Cytochrome b-5, isoform CRA_a (Cytochrome b5)	5310	9500	1.79	Up	10800	2.03	Up	1.14	-
Q61704	ITIH3	Inter-alpha-trypsin inhibitor heavy chain H3 (ITI heavy chain H3) (ITI-HC3) (Inter-alpha-inhibitor heavy chain 3)	2460	2870	1.17	-	3990	1.62	Up	1.39	-
Q8BWM0	PGES2	Prostaglandin E synthase 2 (EC 5.3.99.3) (GATE-binding factor 1) (GBF-1) (Microsomal prostaglandin E synthase 2) (mPGES-2) [Cleaved into: Prostaglandin E synthase 2 truncated form]	24300	15700	0.65	Down	11600	0.48	Down	0.74	-
Q9CQA3	SDHB	Succinate dehydrogenase [ubiquinone] iron-sulfur	4290	2590	0.60	Down	2620	0.61	Down	1.01	-

		subunit, mitochondrial (EC 1.3.5.1) (Iron-sulfur subunit of complex II) (Ip)									
P29788	VTNC	Vitronectin (VN) (S-protein) (Serum-spreading factor)	2310	2770	1.20	-	4480	1.94	Up	1.62	Up
P63276	RS17	40S ribosomal protein S17	2420	3880	1.60	Up	3930	1.62	Up	1.01	-
P10518	HEM2	Delta-aminolevulinic acid dehydratase (ALADH) (EC 4.2.1.24) (Porphobilinogen synthase)	13900	9360	0.67	-	8930	0.64	Down	0.95	-
Q9QZQ8	H2AY	Core histone macro-H2A.1 (Histone macroH2A1) (mH2A1) (H2A.y) (H2A/y)	4710	6520	1.38	-	7790	1.65	Up	1.19	-
Q9D023	MPC2	Mitochondrial pyruvate carrier 2 (Brain protein 44)	994	1430	1.44	-	3510	3.53	Up	2.45	Up
Q9CQX2	CYB5B	Cytochrome b5 type B (Cytochrome b5 outer mitochondrial membrane isoform)	1400	2750	1.96	Up	4100	2.93	Up	1.49	Up
P54797	TNG2	Transport and Golgi organization 2 homolog (Ser/Thr-rich protein T10 in DGCR region)	2950	2110	0.72	-	1830	0.62	Down	0.87	-
Q99LY9	NDUS5	NADH dehydrogenase [ubiquinone] iron-sulfur protein 5 (Complex I-15 kDa) (CI-15 kDa) (NADH-ubiquinone oxidoreductase 15 kDa subunit)	39200	10500	0.27	Down	13000	0.33	Down	1.24	-
Q9Z1P6	NDUA7	NADH dehydrogenase [ubiquinone] 1 alpha subcomplex subunit 7 (Complex I-B14.5a) (CI-B14.5a) (NADH-ubiquinone oxidoreductase subunit B14.5a)	2450	5050	2.06	Up	5080	2.07	Up	1.01	-
P67984	RL22	60S ribosomal protein L22 (Heparin-binding protein HBp15)	762	963	1.26	-	1540	2.02	Up	1.60	Up
Q8BMF3	MAON	NADP-dependent malic enzyme, mitochondrial (NADP-ME) (EC 1.1.1.40) (Malic enzyme 3)	4250	2930	0.69	-	2560	0.60	Down	0.87	-
A2ACG7	A2ACG7	Dolichyl-diphosphooligosaccharide--protein glycosyltransferase subunit 2	5070	5810	1.15	-	7720	1.52	Up	1.33	-
P62960	YBOX1	Nuclease-sensitive element-binding protein 1 (CCAAT-binding transcription factor I subunit A) (CBF-A) (DNA-binding protein B) (DBPB) (Enhancer factor I subunit A) (EFI-A) (Y-box transcription factor) (Y-box-binding protein 1) (YB-1)	449	849	1.89	Up	740	1.65	Up	0.87	-

P32261	ANT3	Antithrombin-III (ATIII) (Serpin C1)	3890	3330	0.86	-	5930	1.52	Up	1.78	Up
A2AUD5	A2AUD5	Tumor protein D54	12200	20400	1.67	Up	19300	1.58	Up	0.95	-
A0A1B0GR19	A0A1B0GR19	Reticulocalbin-3 (Fragment)	5180	8680	1.68	Up	9800	1.89	Up	1.13	-
Q9DCM2	GSTK1	Glutathione S-transferase kappa 1 (EC 2.5.1.18) (GST 13-13) (GST class-kappa) (GSTK1-1) (mGSTK1) (Glutathione S-transferase subunit 13)	13100	6980	0.53	Down	5700	0.44	Down	0.82	-
Q5M8N4	D39U1	Epimerase family protein SDR39U1 (EC 1.1.1.-) (Short-chain dehydrogenase/reductase family 39U member 1)	11200	6140	0.55	Down	6430	0.57	Down	1.05	-
Q62048	PEA15	Astrocytic phosphoprotein PEA-15 (15 kDa phosphoprotein enriched in astrocytes)	4220	3380	0.80	-	2450	0.58	Down	0.72	-
P35564	CALX	Calnexin	12600	9060	0.72	-	6890	0.55	Down	0.76	-
Q61029	LAP2B	Lamina-associated polypeptide 2, isoforms beta/delta/epsilon/gamma (Thymopoietin isoforms beta/delta/epsilon/gamma) (TP beta/delta/epsilon/gamma)	10800	19000	1.76	Up	16900	1.56	Up	0.89	-
P49817	CAV1	Caveolin-1	1770	1630	0.92	-	3660	2.07	Up	2.25	Up
Q8R1I1	QCR9	Cytochrome b-c1 complex subunit 9 (Complex III subunit 9) (Complex III subunit X) (Cytochrome c1 non-heme 7 kDa protein) (Ubiquinol-cytochrome c reductase complex 7.2 kDa protein)	17200	16500	0.96	-	34300	1.99	Up	2.08	Up
Q91V64	ISOC1	Isochorismatase domain-containing protein 1	542	831	1.53	Up	942	1.74	Up	1.13	-
D3YUG3	D3YUG3	40S ribosomal protein S19 (Fragment)	6700	5160	0.77	-	4360	0.65	Down	0.84	-
B1AXW6	B1AXW6	Peroxiredoxin-1 (Fragment)	10700	22200	2.07	Up	20300	1.90	Up	0.91	-
P63325	RS10	40S ribosomal protein S10	4070	5030	1.24	-	6290	1.55	Up	1.25	-
O08600	NUCG	Endonuclease G, mitochondrial (Endo G) (EC 3.1.30.-)	2020	1910	0.95	-	3050	1.51	Up	1.60	Up
Q61768	KINH	Kinesin-1 heavy chain (Conventional kinesin heavy chain) (Ubiquitous kinesin heavy chain) (UKHC)	6920	9880	1.43	-	5270	0.76	-	0.53	Down
Q91ZU6	DYST	Dystonin (Bullous pemphigoid antigen 1) (BPA)	220	397	1.81	Up	348	1.58	Up	0.88	-

		(Dystonia musculorum protein) (Hemidesmosomal plaque protein) (Microtubule actin cross-linking factor 2)								
A2AJI1	A2AJI1	MAP7 domain-containing protein 1	3920	6870	1.75	Up	6430	1.64	Up	0.94	-
Q9JK92	HSPB8	Heat shock protein beta-8 (HspB8) (Alpha-crystallin C chain) (Small stress protein-like protein HSP22)	1160	1180	1.02	-	2860	2.47	Up	2.42	Up
Q99MQ4	ASPN	Asporin (Periodontal ligament-associated protein 1) (PLAP-1)	16600	13200	0.80	-	10200	0.61	Down	0.77	-
Q9D898	ARP5L	Actin-related protein 2/3 complex subunit 5-like protein (Arp2/3 complex 16 kDa subunit 2) (ARC16-2)	606	485	0.80	-	388	0.64	Down	0.80	-
Q62167	DDX3X	ATP-dependent RNA helicase DDX3X (EC 3.6.4.13) (D1Pas1-related sequence 2) (DEAD box RNA helicase DEAD3) (mDEAD3) (DEAD box protein 3, X-chromosomal) (Embryonic RNA helicase)	2840	1090	0.38	Down	1090	0.38	Down	1.00	-
Q9QZ85	IIGP1	Interferon-inducible GTPase 1 (EC 3.6.5.-)	3150	2550	0.81	-	4770	1.51	Up	1.87	Up
Q8C0E2	VP26B	Vacuolar protein sorting-associated protein 26B (Vesicle protein sorting 26B)	3780	4850	1.28	-	7300	1.93	Up	1.51	Up
O09167	RL21	60S ribosomal protein L21	2780	4260	1.53	Up	4890	1.76	Up	1.15	-
P43276	H15	Histone H1.5 (H1 VAR.5) (H1b)	154	406	2.64	Up	484	3.15	Up	1.19	-
P12246	SAMP	Serum amyloid P-component (SAP)	5900	7680	1.30	-	9420	1.60	Up	1.23	-
Q9CR57	RL14	60S ribosomal protein L14	6620	12800	1.93	Up	19900	3.01	Up	1.55	Up
Q9JLZ3	AUHM	Methylglutaconyl-CoA hydratase, mitochondrial (EC 4.2.1.18) (AU-specific RNA-binding enoyl-CoA hydratase) (AU-binding enoyl-CoA hydratase) (muAUH) (Itaconyl-CoA hydratase) (EC 4.2.1.56)	18300	12000	0.66	Down	10500	0.57	Down	0.88	-
P26883	FKB1A	Peptidyl-prolyl cis-trans isomerase FKBP1A (PPIase FKBP1A) (EC 5.2.1.8) (12 kDa FK506-binding protein) (12 kDa FKBP) (FKBP-12) (Calstabin-1) (FK506-binding protein 1A) (FKBP-1A) (Immunophilin FKBP12) (Rotamase)	2570	2960	1.15	-	6650	2.59	Up	2.25	Up

P61358	RL27	60S ribosomal protein L27	2670	3360	1.26	-	5610	2.10	Up	1.67	Up
P01837	IGKC	Immunoglobulin kappa constant (Ig kappa chain C region MOPC 21)	1020	1370	1.34	-	1870	1.83	Up	1.36	-
A0A0R4J0J0	A0A0R4J0J0	Palmdelphin	11000	9810	0.89	-	5020	0.46	Down	0.51	Down
Q6IFX2	K1C42	Keratin, type I cytoskeletal 42 (Cytokeratin-42) (CK-42) (Keratin-17n) (Keratin-42) (K42) (Type I keratin Ka22)	407	570	1.40	-	757	1.86	Up	1.33	-
P05784	K1C18	Keratin, type I cytoskeletal 18 (Cytokeratin endo B) (Keratin D) (Cytokeratin-18) (CK-18) (Keratin-18) (K18)	770	1670	2.17	Up	1640	2.13	Up	0.98	-
Q80YX1	TENA	Tenascin (TN) (Hexabrachion) (Tenascin-C) (TN-C)	503	634	1.26	-	760	1.51	Up	1.20	-
Q60676	PPP5	Serine/threonine-protein phosphatase 5 (PP5) (EC 3.1.3.16) (Protein phosphatase T) (PPT)	757	1290	1.70	Up	1170	1.55	Up	0.91	-
Q61160	FADD	FAS-associated death domain protein (FAS-associating death domain-containing protein) (Mediator of receptor induced toxicity) (Protein FADD)	4050	2650	0.65	Down	2240	0.55	Down	0.85	-
Q921J2	RHEB	GTP-binding protein Rheb (Ras homolog enriched in brain)	3620	2770	0.77	-	4500	1.24	-	1.62	Up
A0A140T8T4	A0A140T8T4	Ribosomal protein L9, pseudogene 6	8890	11600	1.30	-	14100	1.59	Up	1.22	-
O09164	SODE	Extracellular superoxide dismutase [Cu-Zn] (EC-SOD) (EC 1.15.1.1)	1950	2090	1.07	-	3140	1.61	Up	1.50	Up
P82347	SGCD	Delta-sarcoglycan (Delta-SG) (35 kDa dystrophin-associated glycoprotein) (35DAG)	2430	4560	1.88	Up	4850	2.00	Up	1.06	-
P10833	RRAS	Ras-related protein R-Ras (p23)	2590	4840	1.87	Up	5610	2.17	Up	1.16	-
Q8BWY3	ERF1	Eukaryotic peptide chain release factor subunit 1 (Eukaryotic release factor 1) (eRF1)	392	350	0.89	-	252	0.64	Down	0.72	-
Q9CPT4	MYDGF	Myeloid-derived growth factor (MYDGF)	1940	3280	1.69	Up	4770	2.46	Up	1.45	-
Q8BGJ5	Q8BGJ5	MCG13402, isoform CRA_a (Polypyrimidine tract-	692	789	1.14	-	1090	1.57	Up	1.38	-

		binding protein 1)									
Q9CQ91	NDUA3	NADH dehydrogenase [ubiquinone] 1 alpha subcomplex subunit 3 (Complex I-B9) (CI-B9) (NADH-ubiquinone oxidoreductase B9 subunit)	376	355	0.95	-	4810	12.79	Up	13.53	Up
P35980	RL18	60S ribosomal protein L18	4270	6610	1.55	Up	9440	2.21	Up	1.43	-
Q80WJ7	LYRIC	Protein LYRIC (3D3/LYRIC) (Lysine-rich CEACAM1 co-isolated protein) (Metadherin) (Metastasis adhesion protein)	1110	1420	1.28	-	1700	1.53	Up	1.20	-
Q3URE1	ACSF3	Acyl-CoA synthetase family member 3, mitochondrial (EC 6.2.1.-)	1560	1120	0.72	-	1010	0.65	Down	0.90	-
P63005	LIS1	Platelet-activating factor acetylhydrolase IB subunit alpha (Lissencephaly-1 protein) (LIS-1) (PAF acetylhydrolase 45 kDa subunit) (PAF-AH 45 kDa subunit) (PAF-AH alpha) (PAFAH alpha)	3640	3180	0.87	-	4820	1.32	-	1.52	Up
Q62186	SSRD	Translocon-associated protein subunit delta (TRAP-delta) (Signal sequence receptor subunit delta) (SSR-delta)	6820	9990	1.46	-	13300	1.95	Up	1.33	-
Q8C253	Q8C253	Galectin	9510	4970	0.52	Down	6060	0.64	Down	1.22	-
Q60590	A1AG1	Alpha-1-acid glycoprotein 1 (AGP 1) (Orosomucoid-1) (OMD 1)	1380	2050	1.49	-	2530	1.83	Up	1.23	-
P35979	RL12	60S ribosomal protein L12	4990	5770	1.16	-	7790	1.56	Up	1.35	-
Q02013	AQP1	Aquaporin-1 (AQP-1) (Aquaporin-CHIP) (Delayed early response protein 2) (DER2) (Water channel protein for red blood cells and kidney proximal tubule)	141	170	1.21	-	230	1.63	Up	1.35	-
D3Z569	D3Z569	Signal peptidase complex catalytic subunit SEC11 (EC 3.4.21.89)	5460	6600	1.21	-	11100	2.03	Up	1.68	Up
P62309	RUXG	Small nuclear ribonucleoprotein G (snRNP-G) (Sm protein G) (Sm-G) (SmG)	1950	3140	1.61	Up	3930	2.02	Up	1.25	-
Q99K94	Q99K94	Signal transducer and activator of transcription	503	431	0.86	-	696	1.38	-	1.61	Up
Q00519	XDH	Xanthine dehydrogenase/oxidase [Includes: Xanthine	2770	1370	0.49	Down	1720	0.62	Down	1.26	-

		dehydrogenase (XD) (EC 1.17.1.4); Xanthine oxidase (XO) (EC 1.17.3.2) (Xanthine oxidoreductase) (XOR)]									
A0A0B4J1E2	A0A0B4J1E2	SNW domain-containing protein 1	960	1300	1.35	-	1450	1.51	Up	1.12	-
D3YV99	D3YV99	COP9 signalosome complex subunit 4 (Fragment)	3660	2490	0.68	-	1910	0.52	Down	0.77	-
P26516	PSMD7	26S proteasome non-ATPase regulatory subunit 7 (26S proteasome regulatory subunit RPN8) (26S proteasome regulatory subunit S12) (Mov34 protein) (Proteasome subunit p40)	4900	7340	1.50	Up	8050	1.64	Up	1.10	-
O09174	AMACR	Alpha-methylacyl-CoA racemase (EC 5.1.99.4) (2-methylacyl-CoA racemase)	818	913	1.12	-	570	0.70	-	0.62	Down
Q9D020	5NT3A	Cytosolic 5'-nucleotidase 3A (EC 3.1.3.5) (7-methylguanosine phosphate-specific 5'-nucleotidase) (7-methylguanosine nucleotidase) (EC 3.1.3.91) (Cytosolic 5'-nucleotidase 3) (Cytosolic 5'-nucleotidase III) (cN-III) (Lupin) (Pyrimidine 5'-nucleotidase 1) (P5'N-1) (P5N-1) (PN-I)	4660	3380	0.73	-	2760	0.59	Down	0.82	-
P43275	H11	Histone H1.1 (H1 VAR.3) (Histone H1a) (H1a)	956	1410	1.47	-	1500	1.57	Up	1.06	-
Q05816	FABP5	Fatty acid-binding protein 5 (Epidermal-type fatty acid-binding protein) (E-FABP) (Fatty acid-binding protein, epidermal) (Keratinocyte lipid-binding protein) (Psoriasis-associated fatty acid-binding protein homolog) (PA-FABP)	17800	11900	0.67	Down	11200	0.63	Down	0.94	-
P15089	CBPA3	Mast cell carboxypeptidase A (MC-CPA) (EC 3.4.17.1) (Carboxypeptidase A3)	3580	1970	0.55	Down	3270	0.91	-	1.66	Up
Q6PDM2	SRSF1	Serine/arginine-rich splicing factor 1 (ASF/SF2) (Pre-mRNA-splicing factor SRp30a) (Splicing factor, arginine/serine-rich 1)	1340	2070	1.54	Up	2170	1.62	Up	1.05	-
Q8BP67	RL24	60S ribosomal protein L24	2270	3600	1.59	Up	5080	2.24	Up	1.41	-
E9PV66	E9PV66	Myosin XVIIIb	862	794	0.92	-	530	0.62	Down	0.67	Down

Q8R1V4	TMED4	Transmembrane emp24 domain-containing protein 4 (Endoplasmic reticulum stress-response protein 25) (ERS25) (p24 family protein alpha-3) (p24alpha3) (p26)	4760	5790	1.22	-	9210	1.93	Up	1.59	Up
Q9EQI8	RM46	39S ribosomal protein L46, mitochondrial (L46mt) (MRP-L46)	1980	2330	1.18	-	1470	0.74	-	0.63	Down
O09044	SNP23	Synaptosomal-associated protein 23 (SNAP-23) (Syndet) (Vesicle-membrane fusion protein SNAP-23)	2380	4160	1.75	Up	3990	1.68	Up	0.96	-
Q9JIQ3	DBLOH	Diablo homolog, mitochondrial (Direct IAP-binding protein with low pI) (Second mitochondria-derived activator of caspase) (Smac)	526	611	1.16	-	1300	2.47	Up	2.13	Up
Q9CQJ8	NDUB9	NADH dehydrogenase [ubiquinone] 1 beta subcomplex subunit 9 (Complex I-B22) (CI-B22) (NADH-ubiquinone oxidoreductase B22 subunit)	1150	730	0.63	Down	685	0.60	Down	0.94	-
Q8CCK0	H2AW	Core histone macro-H2A.2 (Histone macroH2A2) (mH2A2)	333	617	1.85	Up	714	2.15	Up	1.16	-
Q8R086	SUOX	Sulfite oxidase, mitochondrial (EC 1.8.3.1)	3080	1730	0.56	Down	1890	0.61	Down	1.09	-
P63044	VAMP2	Vesicle-associated membrane protein 2 (VAMP-2) (Synaptobrevin-2)	3800	7220	1.90	Up	8590	2.26	Up	1.19	-
Q60994	ADIPO	Adiponectin (30 kDa adipocyte complement-related protein) (Adipocyte complement-related 30 kDa protein) (ACRP30) (Adipocyte, C1q and collagen domain-containing protein) (Adipocyte-specific protein AdipoQ)	5270	5920	1.12	-	9690	1.84	Up	1.64	Up
Q9D0S9	HINT2	Histidine triad nucleotide-binding protein 2, mitochondrial (HINT-2) (EC 3.-.-.-) (HINT-3)	11700	5770	0.49	Down	5740	0.49	Down	0.99	-
Q9D6K8	FUND2	FUN14 domain-containing protein 2 (Hepatitis C virus core-binding protein 6)	4810	3230	0.67	-	5050	1.05	-	1.56	Up
Q9D5T0	ATAD1	ATPase family AAA domain-containing protein 1 (EC 3.6.1.3) (Thorase)	1160	1310	1.13	-	867	0.75	-	0.66	Down

Q6ZQI3	MLEC	Malectin	2820	3350	1.19	-	4340	1.54	Up	1.30	-
P14847	CRP	C-reactive protein	1620	1770	1.09	-	4170	2.57	Up	2.36	Up
D3YZE7	D3YZE7	Erythroid differentiation-related factor 1 (RIKEN cDNA 2700050L05, isoform CRA_b)	1280	1180	0.92	-	2290	1.79	Up	1.94	Up
P97855	G3BP1	Ras GTPase-activating protein-binding protein 1 (G3BP-1) (EC 3.6.4.12) (EC 3.6.4.13) (ATP-dependent DNA helicase VIII) (GAP SH3 domain-binding protein 1) (HDH-VIII)	6130	4150	0.68	-	3710	0.61	Down	0.89	-
F7B5B5	F7B5B5	Heterogeneous nuclear ribonucleoprotein R	1830	2960	1.62	Up	2990	1.63	Up	1.01	-
Q3UUI3	THEM4	Acyl-coenzyme A thioesterase THEM4 (Acyl-CoA thioesterase THEM4) (EC 3.1.2.2) (Carboxyl-terminal modulator protein) (Thioesterase superfamily member 4)	1950	1580	0.81	-	2450	1.26	-	1.55	Up
G3XA17	G3XA17	Eukaryotic translation initiation factor 4 gamma 2 (Eukaryotic translation initiation factor 4, gamma 2, isoform CRA_e)	1200	969	0.81	-	800	0.67	Down	0.82	-
P62996	TRA2B	Transformer-2 protein homolog beta (TRA-2 beta) (TRA2-beta) (Silica-induced gene 41 protein) (SIG-41) (Splicing factor, arginine/serine-rich 10) (Transformer-2 protein homolog B)	1100	1660	1.51	Up	3200	2.91	Up	1.93	Up
P46471	PRS7	26S proteasome regulatory subunit 7 (26S proteasome AAA-ATPase subunit RPT1) (Proteasome 26S subunit ATPase 2) (Protein MSS1)	4470	1520	0.34	Down	1050	0.23	Down	0.69	-
Q9Z2Z6	MCAT	Mitochondrial carnitine/acylcarnitine carrier protein (Carnitine/acylcarnitine translocase) (CAC) (mCAC) (Solute carrier family 25 member 20)	3770	1810	0.48	Down	2260	0.60	Down	1.25	-
Q00915	RET1	Retinol-binding protein 1 (Cellular retinol-binding protein) (CRBP) (Cellular retinol-binding protein I) (CRBP-I) (mCRBPI)	1520	1900	1.25	-	2350	1.55	Up	1.24	-
A0A087	A0A087	Eukaryotic translation elongation factor 1 beta 2	3720	1560	0.42	Down	1490	0.40	Down	0.96	-

WS46	WS46									
P35282	RAB21	Ras-related protein Rab-21 (Rab-12)	1980	1830	0.92	-	2790	1.41	-	1.52	Up
Q9DC77	SMPX	Small muscular protein (Stretch-responsive skeletal muscle protein) (Chisel protein)	494	610	1.23	-	795	1.61	Up	1.30	-
Q9D6V8	PAIP2	Polyadenylate-binding protein-interacting protein 2 (PABP-interacting protein 2) (PAIP-2) (Poly(A)-binding protein-interacting protein 2)	707	1330	1.88	Up	737	1.04	-	0.55	Down
G3UWC2	G3UWC2	N-acetylated alpha-linked acidic dipeptidase 2, isoform CRA_a (N-acetylated-alpha-linked acidic dipeptidase 2)	1110	1900	1.71	Up	1980	1.78	Up	1.04	-
O35465	FKBP8	Peptidyl-prolyl cis-trans isomerase FKBP8 (PPIase FKBP8) (EC 5.2.1.8) (38 kDa FK506-binding protein) (38 kDa FKBP) (FKBP-38) (mFKBP38) (FK506-binding protein 8) (FKBP-8) (FKBPR38) (Rotamase)	459	1040	2.27	Up	722	1.57	Up	0.69	-
P01921	HB2D	H-2 class II histocompatibility antigen, A-D beta chain	397	337	0.85	-	815	2.05	Up	2.42	Up
A0A1L1SUR7	A0A1L1SUR7	Phosphatidylinositol-binding clathrin assembly protein (Fragment)	1380	2690	1.95	Up	2440	1.77	Up	0.91	-
A0A0A0MQN8	A0A0A0MQN8	Neuroplastin	1600	2210	1.38	-	2470	1.54	Up	1.12	-
P47911	RL6	60S ribosomal protein L6 (TAX-responsive enhancer element-binding protein 107) (TAXREB107)	2480	3830	1.54	Up	5610	2.26	Up	1.46	-
O70591	PFD2	Prefoldin subunit 2	1500	2920	1.95	Up	2430	1.62	Up	0.83	-
P61924	COPZ1	Coatomer subunit zeta-1 (Zeta-1-coat protein) (Zeta-1 COP)	2080	2900	1.39	-	3920	1.88	Up	1.35	-
P56393	COX7B	Cytochrome c oxidase subunit 7B, mitochondrial (Cytochrome c oxidase polypeptide VIIb)	1120	965	0.86	-	3500	3.13	Up	3.63	Up
P50637	TSPO	Translocator protein (Mitochondrial benzodiazepine receptor) (PKBS) (Peripheral-type benzodiazepine receptor) (PBR)	1090	960	0.88	-	1640	1.50	Up	1.71	Up
P62900	RL31	60S ribosomal protein L31	623	879	1.41	-	1080	1.73	Up	1.23	-

P35550	FBRL	rRNA 2'-O-methyltransferase fibrillarin (EC 2.1.1.-) (Histone-glutamine methyltransferase) (Nucleolar protein 1)	1410	525	0.37	Down	584	0.41	Down	1.11	-
P14142	GLUT4	Solute carrier family 2, facilitated glucose transporter member 4 (GT2) (Glucose transporter type 4, insulin-responsive) (GLUT-4)	541	563	1.04	-	1070	1.98	Up	1.90	Up
Q7TQD2	TPPP	Tubulin polymerization-promoting protein (TPPP)	1420	919	0.65	Down	910	0.64	Down	0.99	-
Q91YE8	SYNP2	Synaptopodin-2 (Myopodin)	797	1030	1.29	-	1390	1.74	Up	1.35	-
Q8K4F5	ABHDB	Protein ABHD11 (EC 3.-.-.-) (Alpha/beta hydrolase domain-containing protein 11) (Abhydrolase domain-containing protein 11) (Williams-Beuren syndrome chromosomal region 21 protein homolog)	2060	932	0.45	Down	826	0.40	Down	0.89	-
Q9CXU4	Q9CXU4	Mitochondrial import inner membrane translocase subunit TIM23	1090	2090	1.92	Up	2550	2.34	Up	1.22	-
